# Investigation of regions impacting inbreeding depression and their association with the additive genetic effect for United States and Australia Jersey dairy cattle

**DOI:** 10.1186/s12864-015-2001-7

**Published:** 2015-10-19

**Authors:** Jeremy T. Howard, Mekonnen Haile-Mariam, Jennie E. Pryce, Christian Maltecca

**Affiliations:** Department of Animal Science and Genetics Program, North Carolina State University, Raleigh, NC 27695-7627 USA; Department of Economic Development, Jobs, Transport and Resources and Dairy Futures Cooperative Research Centre, 5 Ring Road, Bundoora, VIC 3083 Australia; La Trobe University, Bundoora, VIC 3086 Australia

## Abstract

**Background:**

Variation in environment, management practices, nutrition or selection objectives has led to a variety of different choices being made in the use of genetic material between countries. Differences in genome-level homozygosity between countries may give rise to regions that result in inbreeding depression to differ. The objective of this study was to characterize regions that have an impact on a runs of homozygosity (ROH) metric and estimate their association with the additive genetic effect of milk (MY), fat (FY) and protein yield (PY) and calving interval (CI) using Australia (AU) and United States (US) Jersey cows.

**Methods:**

Genotyped cows with phenotypes on MY, FY and PY (*n* = 6751 US; *n* = 3974 AU) and CI (*n* = 5816 US; *n* = 3905 AU) were used in a two-stage analysis. A ROH statistic (ROH4Mb), which counts the frequency of a SNP being in a ROH of at least 4 Mb was calculated across the genome. In the first stage, residuals were obtained from a model that accounted for the portion explained by the estimated breeding value. In the second stage, these residuals were regressed on ROH4Mb using a single marker regression model and a gradient boosted machine (GBM) algorithm. The relationship between the additive and ROH4Mb of a region was characterized based on the (co)variance of 500 kb estimated genomic breeding values derived from a Bayesian LASSO analysis. Phenotypes to determine ROH4Mb and additive effects were residuals from the two-stage approach and yield deviations, respectively.

**Results:**

Associations between yield traits and ROH4Mb were found for regions on BTA13, BTA23 and BTA25 for the US population and BTA3, BTA7, BTA17 for the AU population. Only one association (BTA7) was found for CI and ROH4Mb for the US population. Multiple potential epistatic interactions were characterized based on the GBM analysis. Lastly, the covariance sign between ROH4Mb and additive SNP effect of a region was heterogeneous across the genome.

**Conclusion:**

We identified multiple genomic regions associated with ROH4Mb in US and AU Jersey females. The covariance of regions impacting ROH4Mb and the additive genetic effect were positive and negative, which provides evidence that the homozygosity effect is location dependent.

**Electronic supplementary material:**

The online version of this article (doi:10.1186/s12864-015-2001-7) contains supplementary material, which is available to authorized users.

## Background

An individual’s inbreeding coefficient is defined as the probability that any randomly chosen allele at a homologous locus carried by the individual is identical-by-descent (IBD) and equals the coancestry between its parents [1, 2]. Following Wright [1], the inbreeding coefficient for an individual can be calculated and is the expected proportion of the genome that is IBD. The advent of dense single nucleotide polymorphism (SNP) marker panels allows for alternative molecular inbreeding metrics to be estimated. The molecular inbreeding value calculated from the genomic relationship matrix [3, 4] is the probability that the two alleles carried by an individual are identical-by-state (IBS) and is adjusted based on the SNP allelic content [5] and represents the realized proportion of the genome that is homozygous. An alternative way of measuring inbreeding involves genomic runs of homozygosity (ROH). The ROH is a useful measure of inbreeding given its ability to distinguish between chromosome segments that are IBS and IBD. Long ROH segments have low probability of having arisen by chance, and are more likely to be stretches of two homologous chromosomes within the same individual descending from a recent common ancestor [6]. Keller et al. [6] found that ROH based inbreeding estimates are preferable to pedigree derived metrics and other measures of genomic inbreeding, since they correlate strongly with the homozygous mutation load. As a further advantage, ROH measures can be tailored to distinguish between inbreeding arising from a recent common ancestor (longer ROH) or more distant common ancestors (shorter ROH).

High levels of inbreeding result in a reduction in fitness and overall performance at the phenotypic level, due to individuals carrying a large number of deleterious recessive mutations and/or the reduction in frequency of the superior heterozygotes [7]. This reduction is referred to as inbreeding depression and is seen mostly in characters connected with reproductive capacity or physiological efficiency, although any trait under selection can show some degree of inbreeding depression (see [8], for a review). Inbreeding depression is associated with the degree of dominance that exists for a trait and it has been shown that larger negative estimates of inbreeding depression are associated with higher estimates of dominance variance [9]. Furthermore, by constructing founder-specific partial inbreeding coefficients, inbreeding depression has been shown to be heterogeneous across founders [10, 11]. As a consequence, a region of the genome that is derived from an ancestor potentially gives rise to varying levels of inbreeding depression in different progeny. Utilizing dominance as a proxy for characterizing regions that impact inbreeding depression has been utilized previously [12, 13], but is computationally demanding and requires large samples sizes. Recently, alternative ways to characterize inbreeding depression have been proposed. For example in swine [14] and dairy cattle [15], a ROH metric has been utilized to characterize the impact of regions contained within a ROH on economically important traits. The use of genomic information to identify regions that impact inbreeding depression allows for the possibility to distinguish between animals with the same inbreeding coefficient, but that differ in the number of regions that when homozygous cause a reduction in fitness. Additionally, the combination of multiple regions that individually have a minor effect on inbreeding depression, but when combined cause a major reduction in fitness may provide clues as to the previously identified non-linear relationship of inbreeding depression [16]. The use of machine-learning algorithms that utilize regression trees [17] allows for SNP-by-SNP interactions to be characterized and is computationally efficient. Tree based learners have been used previously to identify epistatic interactions between SNP for residual feed intake in dairy cattle [18].

Longer ROH segments (>5 Megabases (Mb)) instead of short (> 0.5 Mb) and moderate (> 1.5 Mb) segments have been shown by simulation to have a higher correlation with the homozygous mutation load when the effective populations size is low (i.e. 100 animals) [6]. This has been confirmed with real data by Pryce et al. [15], who found that longer ROH were associated with a reduction in milk yield that was independent of the proportion of the genome that was homozygous in the Holstein breed. However, the ROH has also been utilized in studies conducted to identify regions that have a high ROH frequency that is most likely due to directional selection [19, 20]. Therefore, it is likely that there are some regions where long stretches of homozygosity have a favorable impact on economically important traits due to the region having undergone strong directional selection based on the additive genetic value of the region, although this has yet to be validated using real data.

Characterizing the homozygosity across the genome and its impact on inbreeding depression in dairy cattle is advantageous due to the large number of cows that are currently being genotyped and the large number of fitness (e.g. fertility) and performance (e.g. milk yield) traits being measured. Specifically, characterizing these regions with the Jersey breed is worthwhile given the higher levels of inbreeding and smaller effective population size when compared to the Holstein breed [21, 22]. Therefore, the first objective is to identify regions that have an impact on inbreeding depression in US and AU Jersey cows using a ROH metric. The second objective is to determine the relationship between additive effects and the ROH status of a SNP.

## Results

### Characterizing regions impacting inbreeding depression

**Fig. 1:**
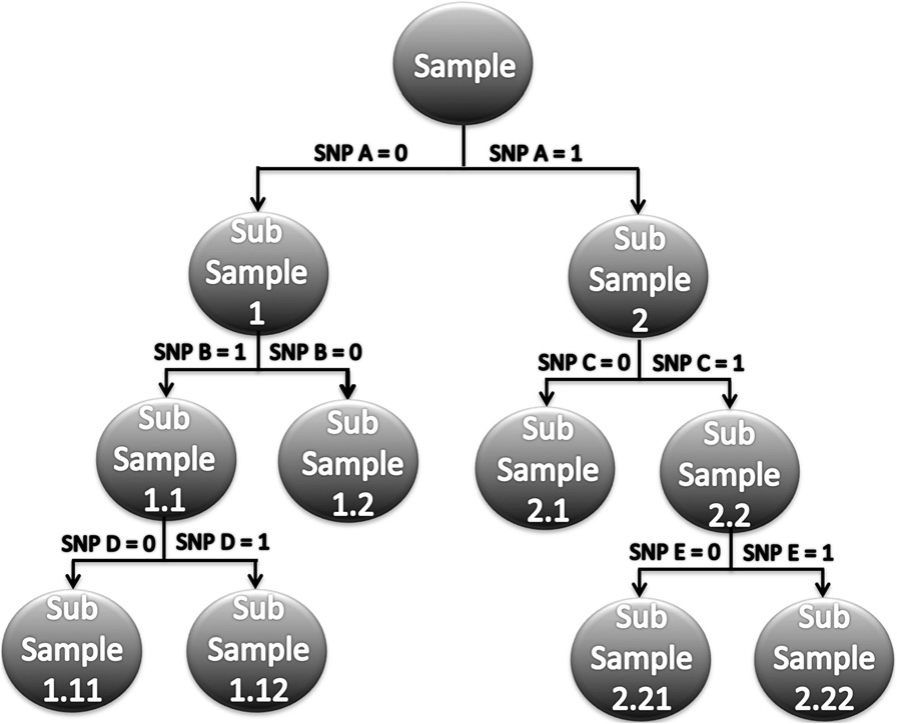
An example of a regression tree generated by Gradient Boosted Machine algorithm based on the run of homozygosity of at least 4 Mb status of a SNP (i.e. 0 or 1). The split point for a particular SNP (i.e. A, B, C, D or E) and the subsample bin an observation falls into based on the genotype value is outlined below each circle. Two SNP that are within the same branch of a tree, such as A-B, A-D, B-D, A-C, A-E and C-E, are referred to as descendent pairs and may indicate epistatic effects and would be tagged as an interaction. The SNP that are not within the same branch of a tree, such as SNP pairs B-C, B-E, D-C and D-E, are referred to as non-descendent pairs and may indicate independent additive genetic effects and not tagged as an interaction

Two cow populations born in the US and AU were utilized to identify regions that when homozygous cause a reduction in milk (MY), fat (FY) and protein yield (PY; *n* = 6751 US; n = 3974 AU) and an increased calving interval (CI; n = 5816 US; *n* = 3905 AU), which is the interval between consecutive calvings and a measure directly linked to fertility. Phenotypic information for the AU population was provided in the form of yield deviations and equivalent variable for US population were calculated after adjusting their phenotypes (e.g. Milk yield) for fixed effects. The ROH status of a SNP (*n* = 31,431) was defined based on whether the SNP was within an ROH of at least 4 Mb in length (ROH4Mb). A two-stage analysis was performed within each population to estimate the effect of the ROH4Mb status of a SNP on milk yield and fertility traits. The first stage involved generating residuals from an animal model that accounted for the additive effects captured by the estimated breeding value (EBV) of the individual. The second stage involved using the residuals from the first stage as a phenotype and regress these on the ROH4Mb status utilizing a single marker regression and gradient boosted machine (GBM). Significance was declared by using a permutation test based on 2500 samples [23]. The identification of epistatic interactions between the ROH4Mb status of a SNP was carried out by counting the number of times two SNP were a descendent pair as described by Yao et al. [18] and outlined in Fig. 1. The significance of the frequency of a descendent pair and variable importance value was then declared based on a permutation test (*n* = 2,500 samples) [23].

The single marker regression and GBM analysis identified multiple regions that have a significant effect when contained within a ROH of at least 4 Mb across multiple traits and populations. A complete list of the regions along with their significance level is outlined in Table 1 for the US and AU populations. Additionally, the negative log of the p-value from the permutation analysis across the genome based on the single marker regression analysis for all traits are presented in Additional file 1: Figure S1 and Additional file 2: Figure S2 for US and AU, respectively. Within a population, the following regions had an effect across multiple traits including BTA13 (19.3–19.9 Mb; MY-PY), BTA23 (32.7–33.3 Mb; MY-FY-PY) and BTA25 (24.8–30.7 Mb; MY-PY) for the US population and BTA3 (113.4–114.6 Mb; FY-PY), BTA7 (6.6–16.7 Mb; FY-PY), BTA17 (68.9–75.0 Mb; MY-FY-PY) for the AU population, although no regions were identified that were significant in both populations. A complete description of genes closest to the SNP with the highest significance based on the single marker regression analysis is outlined in Additional file 3: Table S1.

**Table 1:** Regions of the genome associated with a run of homozygosity of at least 4 Mb for milk and fertility traits across countries

Country^a^	Trait	BTA (Region)^b^	Location^c^	Frequency	*P*-value*	
					Single marker regression	Gradient boosted machine
US	Milk Yield	7 (96.2–96.7)	96,541,131	0.07	0.0005	0.07
		13 (19.3–19.9)	19,388,240	0.10	0.0001	0.02
		23 (32.7–33.3)	32,682,177	0.18	0.0001	0.0019
		25 (24.8–27.5)	25,450,477	0.05	0.00009	0.03
		25 (29.1–29.9)	29,113,430	0.06	0.0009	-
	Fat Yield	8 (82.5–83.4)	83,048,502	0.08	0.0003	0.19
		8 (106.6–107.1)	106,817,894	0.11	0.0002	0.07
		19 (12.7–15.5)	14,409,010	0.07	0.0002	0.005
		20 (34.7–36.3)	36,240,997	0.24	0.0003	0.04
		23 (32.7–33.3)	32,682,177	0.18	0.0003	0.04
	Protein Yield	7 (96.1–96.7)	96,192,503	0.07	0.0002	0.04
		13 (19.3 – 19.5)	19,388,240	0.10	0.0004	0.16
		23 (31.9–33.3)	32,682,177	0.18	0.00008	0.004
		25 (24.8–30.7)	29,113,430	0.06	0.00002	0.02
	Calving Interval	7 (82.1–83.0)	82,173,456	0.09	0.0004	0.003
AU	Milk Yield	17 (72.1–73.5)	73,055,503	0.04	0.00004	0.03
		20 (28.4–29.5)	29,322,034	0.33	0.0001	0.04
	Fat Yield	2 (90.4–91.1)	91,117,564	0.16	0.0004	0.08
		3 (113.8–114.2)	113,930,518	0.06	0.0007	0.20
		7 (6.6–16.7)	8,860,921	0.17	0.00007	0.02
		17 (72.1–75.0)	73,257,794	0.04	0.00002	0.006
		18 (50.8–53.0)	52,024,379	0.15	0.00001	0.005
	Protein Yield	3 (113.4–114.6)	113,845,303	0.06	0.000006	0.02
		7 (8.8–12.8)	8,860,921	0.17	0.0003	0.05
		17 (68.9–75.0)	73,055,503	0.04	0.0000008	0.005
		18 (49.0–52.2)	49,446,631	0.13	0.0005	0.47

^a^AU refers to Australia and US refers to United States

^b^BTA refers to chromosome and the region and location are in Mb build UMD 3.1 (http://bovinegenome.org/cgi-bin/gbrowse/bovine_UMD31/)

^c^Referrs to the location with regions with the highest significance based on Single Marker Regression Analysis

*P-values were generated based on a permutation test (Doerge and Churchill [23]) for each analysis

Multiple regions of the genome were found to display potential interactions based on their frequency as descendent pairs. A complete list is outlined in Table 2. The majority of the significant descendent pairs were associated with at least one SNP that also had a large variable importance score. A gene network analysis was employed to determine if two interacting SNP were within the same network and associations were found including shared protein domain as well genetic interactions.

**Table 2:** Genomic regions that potentially display pairwise epistatic interaction based on the high frequency of it being descendent pair for milk and fertility traits across countries

Country^a^	Trait	SNP 1		SNP 2		Average depth	*P*-value*	Individual rank based on importance score	
		BTA^b^	Location^b^	BTA^b^	Location^b^			SNP 1	SNP 2
US	Milk Yield	23	32,682,177	5	95,459,836	1.18	0.0003	1	5
		30	140,296,904	20	69,528,142	1.24	0.0009	14	30
		19	14,409,010	11	10,271,653	1.24	0.0009	3	46
	Fat Yield	19	41,615,615	19	14,409,010	1.42	0.0002	2	1
		19	41,615,615	2	83,919,557	1.36	0.0006	2	7
		11	56,825,445	5	62,248,841	1.29	0.0006	6	10
		12	12,685,397	7	96,192,503	1.16	<0.001	31	15
	Protein Yield	23	32,682,177	1	24,549,757	1.41	0.0005	1	19
		9	7,645,969	1	24,549,757	1.05	0.0009	65	19
		25	29,428,407	2	113,716,333	1.30	0.0009	2	4
	Calving Interval	7	82,173,456	2	83,616,368	1.53	0.0002	1	3
		25	17,166,118	9	44,951,803	1.18	0.0006	7	2
		26	30,607,485	7	82,173,456	1.15	0.0006	12	1
		7	82,173,456	7	41,207,144	1.43	0.0007	1	5
		8	34,242,903	7	82,173,456	1.43	0.0009	32	1
AU	Milk Yield	22	39,545,402	1	112,497,788	1.03	0.0002	25	14
		21	62,115,138	11	38,445,947	1.04	0.0003	35	47
		14	16,526,322	1	13,304,658	1.27	0.0004	68	17
		20	35,012,179	20	29,322,034	1.46	0.0006	51	3
		22	31,649,896	16	42,262,470	1.32	0.0008	4	41
	Fat Yield	6	56,522,979	2	13,411,225	1.08	0.0002	10	18
		9	59,036,606	8	51,460,409	1.73	0.0005	5	3
		8	51,460,409	7	8,860921	1.26	0.0009	3	4
	Protein Yield	14	38,155,245	7	107,837,688	1.27	0.00007	2	18
		17	38,275,065	17	5,445,294	1.38	0.0005	17	20
		14	38,155,245	8	51,695,384	1.40	0.0006	2	22
		16	64,623,464	11	109,818	1.00	0.0008	42	11
	Calving Interval	24	37,002,274	24	7,380,047	1.04	0.0003	16	44
		5	33,334,061	3	9,686,101	1.38	0.0004	1	3
		15	16,416,329	10	53,560,658	1.25	0.0004	14	6
		17	9,753,430	3	80,517,326	1.40	0.0009	8	27

^a^AU refers to Australia and US refers to United States

^b^BTA refers to chromosome and the region and location are in Mb build UMD 3.1 (http://bovinegenome.org/cgi-bin/gbrowse/bovine_UMD31/)

*P-values were generated based on a permutation test (Doerge and Churchill [23]) for each analysis

### Relationship between additive effect and ROH4Mb status of SNP

We further characterized the relationship between the additive and ROH4Mb effects of a SNP. Estimates of the additive marker effect of each SNP were obtained using a whole genome marker regression on the yield deviations, using the Bayesian LASSO of Park and Casella [24]. The ROH4Mb effect of a SNP was estimated by regressing ROH4Mb of a SNP on the same phenotype as single marker regression and gradient boosted machine and therefore the additive effect explained by the EBV was removed from the phenotype. The relationship between the additive and ROH4Mb status of a region was characterized based on the (co)variance of genomic estimated breeding values (GEBV) based on 500 kb overlapping windows. The 10 largest regions based on their absolute covariance were characterized across all traits and countries.

**Fig. 2:**
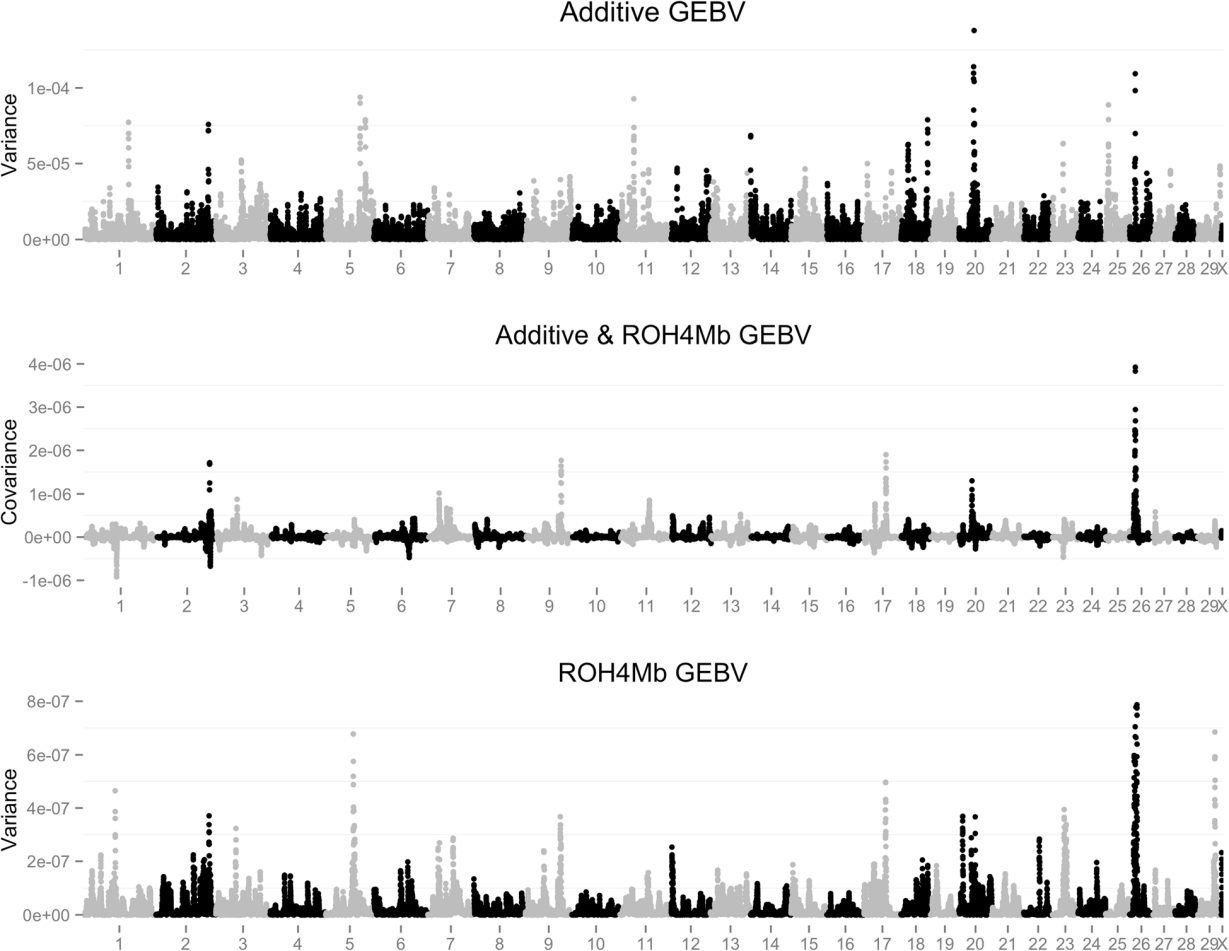
Plot of additive genomic estimated breeding (GEBV) variance, covariance between the additive genomic estimated breeding (GEBV) and ROH4Mb based genomic estimated breeding value and ROH4Mb based genomic estimated breeding value variance across the genome for milk yield on the United States dataset. The region from 1.5 to 2.3 Mb on BTA14 were removed surrounding the DGAT mutation in order to make visualization more informative

**Fig. 3:**
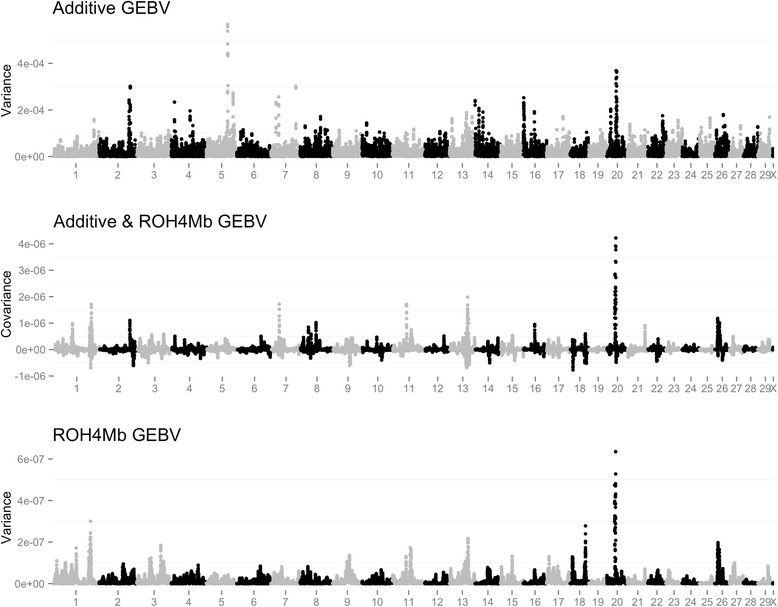
Plot of additive genomic estimated breeding (GEBV) variance, covariance between the additive genomic estimated breeding (GEBV) and ROH4Mb based genomic estimated breeding value and ROH4Mb based genomic estimated breeding value variance across the genome for milk yield on the Australian dataset. The region from 1.5 to 2.3 Mb on BTA14 were removed surrounding the DGAT mutation in order to make visualization more informative

The relationship between the additive effect, ROH4Mb effect and their covariance is outlined for MY in Figs. 2 and 3 for US and AU, respectively. The remaining traits are outlined in Additional file 4: Figure S3, Additional file 5: Figure S4, Additional file 6: Figure S5, Additional file 7: Figure S6, Additional file 8: Figure S7 and Additional file 9: Figure S8 for FY, PY and CI across both countries. As illustrated by Figs. 2 and 3, the covariance sign between ROH4Mb status and the additive effect of a SNP is heterogeneous across the genome. Regions on BTA3 (47.25–54.15), BTA7 (24.26 – 49.00), BTA20 (23.71–34.98) and BTA26 (9.34 – 20.71) had a positive covariance between the additive and ROH4Mb effect of a SNP across both populations. It is worth noting that although there are regions with high ROH4Mb across both populations this does not necessarily imply that they are the same (i.e. IBD) run of homozygosity, instead just confirms that across both populations the region has a similar relationship with the additive genetic value of the individual. Furthermore, within these regions the estimate of the ROH4Mb effect was positive for the majority of the regions, such that it is beneficial for a SNP to be within a long stretch of homozygosity likely resulting from the region having undergone strong directional selection, which was confirmed by Howard et al. [19]. The majority of the regions with the largest absolute covariance value across traits were positive, which is not surprising due to a low frequency of ROH4Mb status for regions with a large ROH4Mb effect (mean ROH4Mb frequency = 0.089) in comparison to the regions that displayed a large positive covariance (mean ROH4Mb frequency = 0.235).

## Discussion

### Characterizing regions impacting inbreeding depression

A single marker regression and an ensemble learning method, GBM, were utilized in the current analysis to characterize regions that have an impact on inbreeding depression based on a ROH metric. In order to determine how similar the results were for the single marker regression and GBM analysis, a rank correlation based on SNP used across both analysis was estimated within each trait and population. The variables utilized in the correlation were the significance value for single marker regression and the variable importance score for GBM. The rank correlation across traits and population ranged from 0.48 to 0.65. A rank correlation of less than unity is not surprising given that the importance score from the GBM analysis captures both the ROH4Mb effect of the SNP and its epistatic interactions with all other SNP.

There were no regions in common across the USA and AU populations that had a significant impact on inbreeding depression. Interbull correlations [25] of EBVs between the US and AU are below unity (ranging from 0.75 and 0.80 for milk, fat and protein yield), indicating that at the additive genetic level, a genotype by environment interaction exists. The equivalent for non-additive effects is unknown, however current results suggest the potential for a genotype by environment interaction to exist at the non-additive genetic level too. Alternative reasons for the lack of concordance between the two populations may be due to a different population history, resulting from a different set of founder sires and or breeding objectives. The introgression of US germplasm in AU genetics is relatively recent and occurred around 20 years ago [22], therefore it is not surprising that the two populations might have a different set of influential sires. An alternative method to characterize the genetic differences across the population is to conduct a principal component analysis on the genomic relationship matrix. A principle component analysis was conducted previously by Howard et al. [19] on a subset of the cows utilized in the current study and the variance explained by the first principle component was 0.024, which illustrates slight differences across the populations.

In order to determine if regions obtained from the two-stage analysis show some degree of dominance using the actual (raw or unadjusted) phenotype an estimate of the additive and dominance effect of SNP declared as being significant were estimated. Actual phenotypes were only available for the US dataset and therefore only SNP that were declared significant within the US population were utilized. The dominance *p*-value across all SNP for the US population was below 0.1 for 10 out of 15 SNP (66.7 %), although only 3 SNP would be significant at the 0.05 level based on the Bonferroni multiple correction factor. In our analysis, the residuals from the 2 stage analysis were corrected for the individuals’ EBV. It is worth noting though that some of the residual may still contain an additive genetic component, thus creating a partial confounding in subsequent analyses. The average accuracy of the estimated breeding values (EBV) in our data was 0.76 and 0.42 for PY and CI, suggesting that the EBV correction does provide a reasonably good measure of the additive breeding value of the individual. In order to confirm that additive effects from SNP information were not within the residual from the two-stage analysis, the correlation between yield deviations that were corrected for the additive SNP effects estimated from the LASSO model and the residual from the two-stage were computed for the US population. The correlations between the two measures for protein yield and calving interval was 0.94 and 0.99 and is displayed graphically in Additional file 10: Figure S9. Based on the dataset used in the current study the two-stage approach provided a more flexible and computationally less demanding way to investigate inbreeding effects, although these regions need to be validated in other populations and fine mapped to identify the possible gene/causative mutations.

The animals used in the study had multiple production records and therefore the regions characterized in the currently study result in a reduction in performance and/or fitness of the animal, but are not lethal or individually do not have a large impact on fitness. Furthermore, individuals that are homozygous in these regions actually have a chance of being allowed to stay in a herd, unlike when lethal or large effect mutations exist, due to the animal having a small likelihood of being born alive. Furthermore, it has been argued that the ability for a population to purge deleterious homozygous mutations is greater for large effect and/or lethal mutations and is not as effective for mutations with minor effects [26]. From this the need to introduce alternative metrics that characterize the effect of region-specific stretches of homozygosity and are less reliant on the assumption that two individuals with the same inbreeding value, also have the same level of inbreeding depression. The use of functional inbreeding metrics that allow for the identification of individuals that are homozygous in areas that have no impact on an economical trait has the potential to allow for greater flexibility in managing herds/populations at the genomic level. One such example could be based on region-specific measures of the effect of homozygosity in order to generate the expected reduction in performance due to inbreeding and/or the probability of being culled at a given parity. The effectiveness of using regions specific inbreeding effects to minimize inbreeding depression while maximizing the genetic gain in a population has yet to be fully understood and should be considered in future research. Additionally, the use of ROH based metrics to manage population diversity and curb inbreeding depression could have even greater potential in small population due to a lack of power in these populations to estimate the dominance effect, using the more traditional metrics.

We identified regions with a putative multiplicative effect for all traits. A further network analysis was able to identify networks shared between two interacting SNP. The existence of ROH4Mb by ROH4Mb interaction between two loci would generate non-linear epistasis in the form of diminishing (reduction in performance is less than the sum of the individual effects) or reinforcing (reduction in performance is greater than sum of the individual effects) epistasis [27]. A few regions that displayed interactions based on the GBM analysis were found to share network associations. To determine if the descendent pairs resulted from a dominance by dominance interaction, a traditional parametric linear model was fitted using the actual phenotype as a response variable that included both SNP additive and dominance effects and their interactions. None of the dominance-by-dominance interactions terms were significant across SNP interactions. It should be noted though that linear models are greedier than non-parametric models and that a larger number of observations might be needed in this case to confirm potential associations. Albeit the ability to detect ROH4Mb by ROH4Mb interaction in the current population is relatively low given the small number of animals our analysis provides a blueprint that can be easily replicated, and as the number of genotyped cows increases this approach could become more powerful.

### Relationship between the additive and inbreeding depression effect of region

The relationship between the additive and ROH4Mb effects based on their GEBV covariance was characterized across the genome for multiple traits. The regions on BTA3 (47.25–54.15Mb), BTA7 (24.26 – 49.00Mb), BTA20 (23.71–34.98Mb) and BTA26 (9.34 – 20.71Mb) have been previously found to be under positive directional selection [19, 28–32]. It should be pointed out that this analysis as described in the previous section could be hampered by the fact that a portion of the additive genetic value might still be contained in the residuals, thus causing a positive covariance. The region on BTA7 has been found in multiple selection signature studies across a variety of cattle breeds such as Jersey (19,30), Angus (30), Nellore cattle [31] and Fleckvieh [32]. The region is gene dense with multiple olfactory receptors, which detect and identify a wide range of odors, providing a cue for the animal to interact with its environment. The region on BTA20 contains the growth hormone receptor gene (*GHR*), which has been associated with milk yield and composition [33] and has been shown previously to be under positive selection [19, 30]. The positive covariance between the additive and ROH4Mb effect for regions most likely undergoing positive directional selection is expected to be due to the favorable allele(s) being driven towards fixation and therefore in this situation homozygosity at this particular region is beneficial to the animal. It is expected that regions that reduce the fitness of the organism to be at a low frequency, which was seen in the current study. Due to this the majority of the population does not have the haplotype therefore the ability to precisely estimate the covariance is limited. This may partly explain the fact that the majority of regions with the largest absolute covariance value across traits were also beneficial.

With the current study we have shown that regions that impact inbreeding depression are variable across populations. The causes of this heterogeneity are manifold: potential lack of power, from a different number of founder individuals, to varying numbers of influential sires in the previous generations, different mating programs or selective goals over time. Interestingly in the current study the majority of the regions identified were at a low frequency. This is important since the power to estimate effects when the frequency of the region is low is reduced and most regions of small effects would be missed by only using a single population. Due to this, a population may not show an effect based on the current set of animals utilized, but over time the frequency may increase and the power will be sufficient to estimate it. Therefore if a region has been shown to be sensitive to long stretches of homozygosity in other populations then long stretches of homozygosity in a population under study should be further investigated. This is also one of the primary limitations of using medium density genomic data, given that the majority of variants impacting inbreeding depression are probably carried at low frequency and these would be in low LD with the ones used in SNP assays. Nevertheless, identifying regions across multiple countries capitalize on the fact the ROH regions differ across populations as confirmed by Howard et al. [19] using the same populations.

Furthermore, the *a priori* knowledge on the impact of a region when it is contained within a long stretch of homozygosity can be utilized in mating schemes in order to constrain homozygosity at specific regions while allowing homozygosity at other regions. Previous research has used methods that constrain relationships averaged across the genome [34–38], although this study confirms that the effect of a region on inbreeding depression is dependent on the genomic region and more importantly some regions are advantageous when homozygous.

## Conclusion

Genomic regions across multiple traits were found to be associated with ROH4Mb on BTA13, BTA23 and BTA25 for the US population and BTA3, BTA7, BTA17 for the AU population. Furthermore, multiple potential epistatic interactions were characterized. The regions on BTA3, BTA7, BTA20 and BTA26 displayed a large positive covariance between the ROH4Mb and the SNP effect and these regions have been previously found in signatures of selection studies. This provides evidence that the effect of a region being homozygous is dependent on the genomic location. Future work should investigate the effectiveness of incorporating location specific inbreeding effect into mating designs using simulated and real data.

## Methods

### Data

No animal care approval was required for the present manuscript because all records came from field data. Phenotype and pedigree information on US Jersey and AU Jersey cows were provided by the American Jersey Cattle Association (Reynoldsburg, OH) and the Australian Dairy Herd Improvement Scheme (ADHIS; Melbourne, Australia), respectively. For the US dataset, the phenotypes used were the same in both populations and included standardized 305 day lactation milk (MY), fat (FY) and protein yield (PY) and calving interval (CI; a measure of fertility). For the AU dataset, yield deviations were already estimated and in order to make comparisons similar yield deviations were constructed for the US population that was based on the same model using ASReml [39], as outlined below:

y_ijklm_ = μ + HYS_i_ + parity_j_ + month_k_ + age + e_ijklm_ (Model 1)

where y_ijklm_ refers to either standardized MY, FY, PY, or CI, μ is the intercept, HYS_i_ is the fixed effect of herd-year-season of calving, parity_j_ was the fixed effect of parity, month_k_ was the fixed effect of month of calving, and age was the regression of age at first calf. Residuals were the only random effects in the model. For cows with multiple lactation records, the average of yield deviations generated from Model 1 were used. Yield deviations were standardized to have a mean of 0 and a variance of 1 to ensure that the results were not affected by systematic differences between the two populations.

Genotypic information on US Jersey cows (*n* = 8235) and AU Jersey cows (*n* = 4075) were provided the American Jersey Cattle Association (Reynoldsburg, OH) and the Australian Dairy Herd Improvement Scheme (ADHIS; Melbourne, Australia), respectively. A complete description of the SNP panels used and SNP editing is outlined by Howard et al. [19]. Briefly, genotype quality control was applied within the US and AU populations separately and consisted of removing animals that had less than 90 % of the SNP called, SNP with a minor allele frequency (MAF) below 0.01 and a p-value of a chi-square test for Hardy-Weinberg equilibrium less than 0.001. Missing SNP were imputed using Beagle [40] and SNP with an imputation accuracy (i.e. Beagle r^2^) of less than 97.5 % were removed. The SNP that passed quality control and were in common across the two populations (*n* = 31,431 SNP) were used for the analysis.

The ROH metric outlined by Kim et al. [20] was used to declare if a SNP was in a ROH. A sliding window approach with a fixed Megabase (Mb) length was used to define ROH regions and a ROH was declared when a region of at least 4 Mb contained only contiguous homozygous SNP with no heterozygotes observed. The sliding window approach started with the first SNP on a chromosome and combined all SNP within 4 Mb into a window and ROH status declared then the window was shifted by one SNP to form a new window that was at least 4 Mb and this process was repeated until the end of a chromosome. The 4 Mb threshold was chosen because it has been shown that the medium density SNP panel is not sensitive enough for the precise determination of short ROH segments [41]. The ROH status of a SNP was defined as whether the SNP was within a ROH of at least 4 Mb in length (ROH4Mb). The ROH4Mb of a SNP was tagged as 1 if the SNP was in a ROH and 0 otherwise.

### Statistical models

A two-stage analysis was performed within each population to estimate the effect of the ROH4Mb status of a SNP on milk yield and fertility traits as outlined by Gulisija et al. [16]. The first stage involved generating residuals from an animal model that accounted for the additive genetic effects. The second stage involved using the residuals from the first stage as a phenotype and regress phenotype on ROH4Mb status. As inbreeding depression is expected to be a function of dominance effects and interactions involving dominance effects [7], this method should mean that residuals derived from the first stage are free of additive genetic effects captured by the estimated breeding value of the individual. An alternative model was investigated that fit additive and dominance effects simultaneously utilizing a subset of the data. The SNP that had a large dominance effect was also found to display a large effect based on the ROH4Mb status using the two-stage approach. Therefore, the two-stage approach was utilized due to greater computational flexibility and possibility to explore s various models such as single-marker regression and nonparametric methods that allow for the detection of interaction terms.

Across both stages, only animals that had both phenotypic and genotypic information for milk production traits (*n* = 6751 US; *n* = 3974 AU) and CI (*n* = 5816 US; *n* = 3905 AU) were used.

#### Stage one

Residuals of a model that accounted for the additive genetic effects captured by the estimated breeding value of the individual were obtained using the following animal model in ASReml [39]:

$$ {\mathrm{y}}_{\mathrm{ijk}}=\upmu +{\mathrm{u}}_{\mathrm{k}}+\frac{{\mathrm{e}}_{\mathrm{ijk}}}{{\mathrm{w}}_{\mathrm{ijk}}} $$ (Model 2)

where y_ijk_ refers to the yield deviation for MY, FY, PY, or CI and *μ* is the intercept. Random effects included u_k_ the additive genetic effect of the k^th^ individual assumed ~ N (0, **A**), with **A** representing the additive relationship matrix derived from a pedigree that traced back at least 4 generations. The pedigree was constructed based on a recursive algorithm to compute the inverse of A assuming a non-inbred population [42]. The algorithm used to construct **A** was based on section 4.3 in Henderson [42], which allows for **D** (i.e. diagonal of the L matrix) to take on only three values $\sqrt{.5},\sqrt{.75}$ and 1 if both parents are known, only one parent is known or no parents are know, respectively. This was done in order to allow for the residuals to retain the portion that was due to inbreeding that would have been accounted for when **A** was constructed based on the Meuwissen and Luo [43] algorithm in ASReml. The random residual, e_ijk_, was weighted by w_ijk_ for the k^th^ individual according to Garrick et al. [44] to account for the fact that individuals may have multiple yield deviation records. The formula used to calculate w_ijk_ was:

$$ \frac{\left(1-{h}^2\right)}{h^2+\frac{1+{r}^2\left(l-1\right)}{l}-{h}^2}, $$

where *h*^*2*^ refers to the heritability, *r*^*2*^ refers to the repeatability and *l* refers to the number of records. The h^2^ and r^2^ values used for all three milk yield traits were 0.25 and 0.43, respectively. The h^2^ and r^2^ values used for CI were 0.08 and 0.17, respectively.

#### Stage two

##### Single marker regression

A single marker regression approach was applied using the following model:

y_ij_ = μ + ROH4Mb_j_ + e_ij_ (Model 3)

where y_ij_ refers to the yield deviation for MY, FY, PY, or CI and μ is the intercept, ROH4Mb_j_ is the ROH4Mb status for SNP_j_ and e_ij_ is the random residual. One of the drawbacks with GWAS is deriving the correct threshold to use, so that the number of false positives arising through multiple testing is minimized. Our approach was to use a permutation test to empirically derive a statistical threshold and consequently reduce the number of false-positives due to multiple hypotheses being tested [23]. A permutation sample was constructed by randomly shuffling the phenotypes, while leaving the ROH4Mb status the same and rerunning Model 3. The process was repeated 2500 times to obtain a distribution of random false positives. Significance was reported as the number of times the observed test statistic was greater than a permutation sample test statistic across all SNP. Regions that had at least 3 contiguous significant SNP were declared significant.

##### Gradient boosting machine

Machine learning algorithms such as gradient boosting machines (GBM) that generate a decision tree provide a convenient and computationally efficient way to explore high order interactions. We used GBM to explore the degree of ROH4Mb by ROH4Mb interaction that occurs across traits. The GBM algorithm, which was introduced by Friedman [45], produces an ensemble of regression tree predictors and each individual tree is grown to a user-specified number of splits [17]. A decision tree generated from the GBM algorithm, as illustrated in Fig. 1, partitions the space of input variables by splitting the observations into homogenous quadrants and each tree split corresponds to an if-then rule for a predictor variable. At each split point in each tree, a different subset of SNP predictor variables (i.e. ROH4Mb status of SNP) is evaluated to determine the best SNP for splitting. This structure of a decision tree naturally encodes and models the interactions between predictor variables [46]. Previous research has shown that GBM performs as well or better than the more popular random forest (RF) and it has a much lower computational burden compared to RF [15, 47]. A description of the algorithm can be found in Friedman [45] and a review by Natekin and Knoll [46]. Briefly, a decision tree such as the one illustrated in Fig. 1 is grown by splitting the sample into two parts, referred to as “daughter nodes”, based on the ROH4Mb value (i.e. 0 or 1). The criterion to select a SNP and its split point is to achieve the best increase in homogeneity in the daughter nodes by minimizing a loss function. For each iteration of the GBM algorithm, a small tree is added to the model as a predictor followed by searching for the next tree that optimally reduces the residual [47]. The variable importance measure, which is based on the number of times a variable is selected for splitting regardless of the interaction depth, is then used to assess the importance of a SNP on a given phenotype [47].

In the current study, the “gbm” R package [48] was used to carry out the analysis within each population and trait. The SNP that had a p-value from the permutation test of less than 0.10 for the single marker regression analysis were used as predictor variable in order to reduce computational time. A Gaussian distribution was assumed across all analysis and a 4 fold cross-validation was used to determine the optimal number of trees to construct, interaction depth and shrinkage. The shrinkage parameter minimizes the degree of overfitting of the model. The optimal parameter was chosen by starting with high and low values for each parameter and either increasing or decreasing them until the minimum mean square error was reached. The final model for all traits based on minimizing the mean squared error was constructed from 1200 trees at an interaction depth of 5 and a shrinkage parameter of 0.0075. It has been shown that linkage disequilibrium introduces a bias in the relative importance measure due to a correlation among predictor variables [49]. In order to reduce the correlation among predictor variables, within each chromosome if the correlation between SNP, based on ROH4Mb status, exceeded 0.1 as outlined by Lubke et al. [47] and only the SNP with the largest impact based on the single marker regression analysis was kept for the final analysis. The final number of SNP utilized for milk production traits and fertility was 115 and 81 for the US dataset and 100 and 105 for the AU dataset, respectively.

The identification of epistatic interactions between the ROH4Mb status of a SNP was carried out using the methodology outlined by Yao et al. [18]. Based on Fig. 1, assume SNP B and D have a large epistatic interaction on a trait. The SNP pairs are represented based on the levels at which they appear, such that SNP D was derived from a split produced by SNP B and therefore represent a parent (i.e. SNP B) and child (i.e. SNP D) descendent pair. The SNP B and D will appear more frequently in the same branch of a tree due to the pair having an epistatic interaction. The lower level descendent pair such as parent (i.e. A) grandchild (i.e. D), will also be referred to as a descendent pair. Therefore, the level of the interaction (i.e. 2-way, 3-way, etc.) is not explicitly generated. Based on the tree generated in Fig. 1, adding SNP D reduces the residual conditionally on the split produced by its ancestor, which appears at a higher-level branch [18]. The identification of SNP with independent effects, such as SNP B and C will also appear frequently within the trees, but they won’t be tagged as descendent pairs due to SNP B and C being on separate branches. Based on this approach, the frequency of a descendent pair across all trees was tabulated for each trait and population. The number of levels that separate two descendent pairs was also tabulated in order to give an idea of whether the descendent pairs occurred more frequency as a parent-child or parent-grandchild. For example based on Fig. 1, the number of levels that separate SNP A and B is 1 and is 2 for SNP A and D.

The significance of the frequency of a descendent pair and variable importance value was declared based on a permutation test (*n* = 2,500 samples) [23]. Within each population the phenotypes were shuffled while the ROH4Mb status remained unchanged and the GBM algorithm and tabulating the frequency of a descendent pair was repeated for each sample. Significance was reported as the number of times the observed variable importance value or descendent pair frequency was greater than the permutation sample across all SNP.

##### Relationship between additive and ROH4Mb status SNP Effects

In order to determine the relationship across the genome for the additive genetic effect and ROH4Mb status, the Bayesian LASSO of Park and Casella [24] was used to estimate all SNP effects simultaneously. The LASSO algorithm was used due to its shrinkage properties and the mean rank correlation across the subset of SNP utilized in the GBM algorithm between single marker, GBM and the LASSO analysis was 0.60. For the LASSO analysis that estimates the additive effect of a SNP, the genotypes were coded as 0 for the homozygote, 2 for the other homozygote and 1 for the heterozygote. Yield deviations from Model 1 were used as phenotypes. An analysis that captures the inbreeding effects based on ROH4Mb status of a SNP was conducted based on the residuals of Model 2 as phenotypes. The LASSO analysis was performed using the ‘BLR’ package in R [50]. A total of 800,000 iterations were run with the first 200,000 discarded as burn-in and a thinning rate of 50. Convergence was checked using the ‘coda’ package [51] by constructing trace plots. To characterize the relationship between the additive genetic value of a SNP and its impact on inbreeding depression across the genome 500 kb, overlapping windows were used to estimated the GEBV variance for a given window for both analysis. The covariance was estimated to determine the direction of the relationship between the two. Then the 10 largest regions based on their absolute covariance were characterized across all traits and countries. The covariance was used instead of the correlation due to unstable correlations due to a small denominator term when computing the correlation.

##### Annotation

Regions that had contiguous significant (*P*-value < 0.001) SNP based on the single marker regression and had a significant SNP-by-SNP interaction for the GBM analysis were investigated further using cow positional candidate genes using Bos Taurus assembly (UMD3.1; Ensemble 78) for functional characterization. Candidate genes were chosen based on their location relative to the SNP with the largest significance. Furthermore, a gene network work analysis was undertaken using GeneMANIA [52] in order to identify pathways that are in common across genes within regions that were examined further.

## Additional files

Additional file 1: Figure S1. The significance^1^ across all traits for the United States population based on Single Marker Regression Analysis. (DOC 618 kb) Additional file 2: Figure S2. The significance^1^ across all traits for the Australia population based on Single Marker Regression Analysis. (DOC 572 kb) Additional file 3: Table S1. Chromosomal locations and candidate genes associated with inbreeding depression for milk and fertility traits across countries. (DOC 45 kb) Additional file 4: Figure S3. Plot of additive genomic estimated breeding (GEBV) variance, covariance between the additive genomic estimated breeding (GEBV) and ROH4Mb based genomic estimated breeding value and ROH4Mb based genomic estimated breeding value variance across the genome for fat yield on the United States dataset. The region from 1.5 to 2.3 Mb on BTA14 were removed surrounding the DGAT mutation in order to make visualization more informative. (DOC 400 kb) Additional file 5: Figure S4. Plot of additive genomic estimated breeding (GEBV) variance, covariance between the additive genomic estimated breeding (GEBV) and ROH4Mb based genomic estimated breeding value and ROH4Mb based genomic estimated breeding value variance across the genome for fat yield on the Australian dataset. The region from 1.5 to 2.3 Mb on BTA14 were removed surrounding the DGAT mutation in order to make visualization more informative. (DOC 486 kb) Additional file 6: Figure S5. Plot of additive genomic estimated breeding (GEBV) variance, covariance between the additive genomic estimated breeding (GEBV) and ROH4Mb based genomic estimated breeding value and ROH4Mb based genomic estimated breeding value variance across the genome for protein yield on the United States dataset. (DOC 419 kb) Additional file 7: Figure S6. Plot of additive genomic estimated breeding (GEBV) variance, covariance between the additive genomic estimated breeding (GEBV) and ROH4Mb based genomic estimated breeding value and ROH4Mb based genomic estimated breeding value variance across the genome for protein yield on the Australian dataset (DOC 397 kb) Additional file 8: Figure S7. Plot of additive genomic estimated breeding (GEBV) variance, covariance between the additive genomic estimated breeding (GEBV) and ROH4Mb based genomic estimated breeding value and ROH4Mb based genomic estimated breeding value variance across the genome for calving interval on the United States dataset. (DOC 449 kb) Additional file 9: Figure S8. Plot of additive genomic estimated breeding (GEBV) variance, covariance between the additive genomic estimated breeding (GEBV) and ROH4Mb based genomic estimated breeding value and ROH4Mb based genomic estimated breeding value variance across the genome for calving interval on the Australian dataset. (DOC 500 kb) Additional file 10: Figure S9. Relationship between yield deviations that were corrected for the additive SNP effects estimated from the LASSO model and the residuals form the two stage analysis for protein yield and calving interval for the US population. (DOC 252 kb)

## Abbreviations

ROH: Runs of homozygosity
MY: Milk yield
FY: Fat yield
PY: Protein yield
CI: Calving interval
AU: Australia
US: United States
ROH4Mb: ROH of 4 Megabases
GBM: Gradient boosted machine
IBD: Identical-by-descent
SNP: Single nucleotide polymorphism
IBS: Identical-by-state
EBV: Estimated breeding value
GEBV: Genomic estimated breeding value
Mb: Megabase
