# Supplementary material for: Investigation of regions impacting inbreeding depression and their association with the additive genetic effect for United States and Australia Jersey dairy cattle
Source: BMC Genomics. 2015 Oct 19;16:813. doi: 10.1186/s12864-015-2001-7 (PMC4612420; doi:10.1186/s12864-015-2001-7)
Supplement: Additional file 1: Figure S1. — The significance1 across all traits for the United States population based on Single Marker Regression Analysis. (DOC 618 kb) [file 12864_2015_2001_MOESM1_ESM.doc]

**Figure S1.** The significance^1^ across all traits for the United States population based on Single Marker Regression Analysis.

**
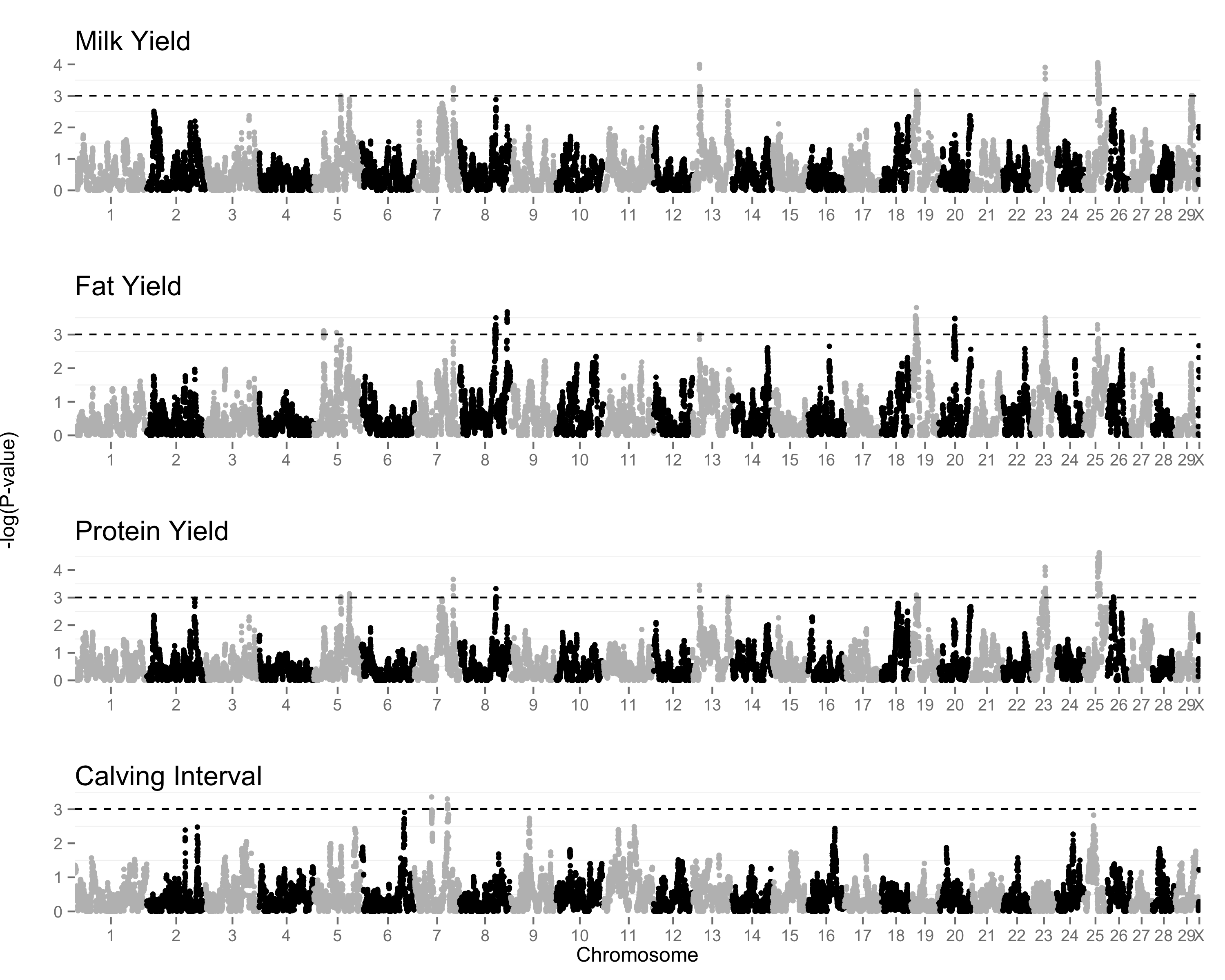
**

**^1^** Dotted lines represent the significance cutoff value.
