# Supplementary material for: Investigation of regions impacting inbreeding depression and their association with the additive genetic effect for United States and Australia Jersey dairy cattle
Source: BMC Genomics. 2015 Oct 19;16:813. doi: 10.1186/s12864-015-2001-7 (PMC4612420; doi:10.1186/s12864-015-2001-7)
Supplement: Additional file 2: Figure S2. — The significance1 across all traits for the Australia population based on Single Marker Regression Analysis. (DOC 572 kb) [file 12864_2015_2001_MOESM2_ESM.doc]

**Figure S2.** The significance^1^ across all traits for the Australia population based on Single Marker Regression Analysis.


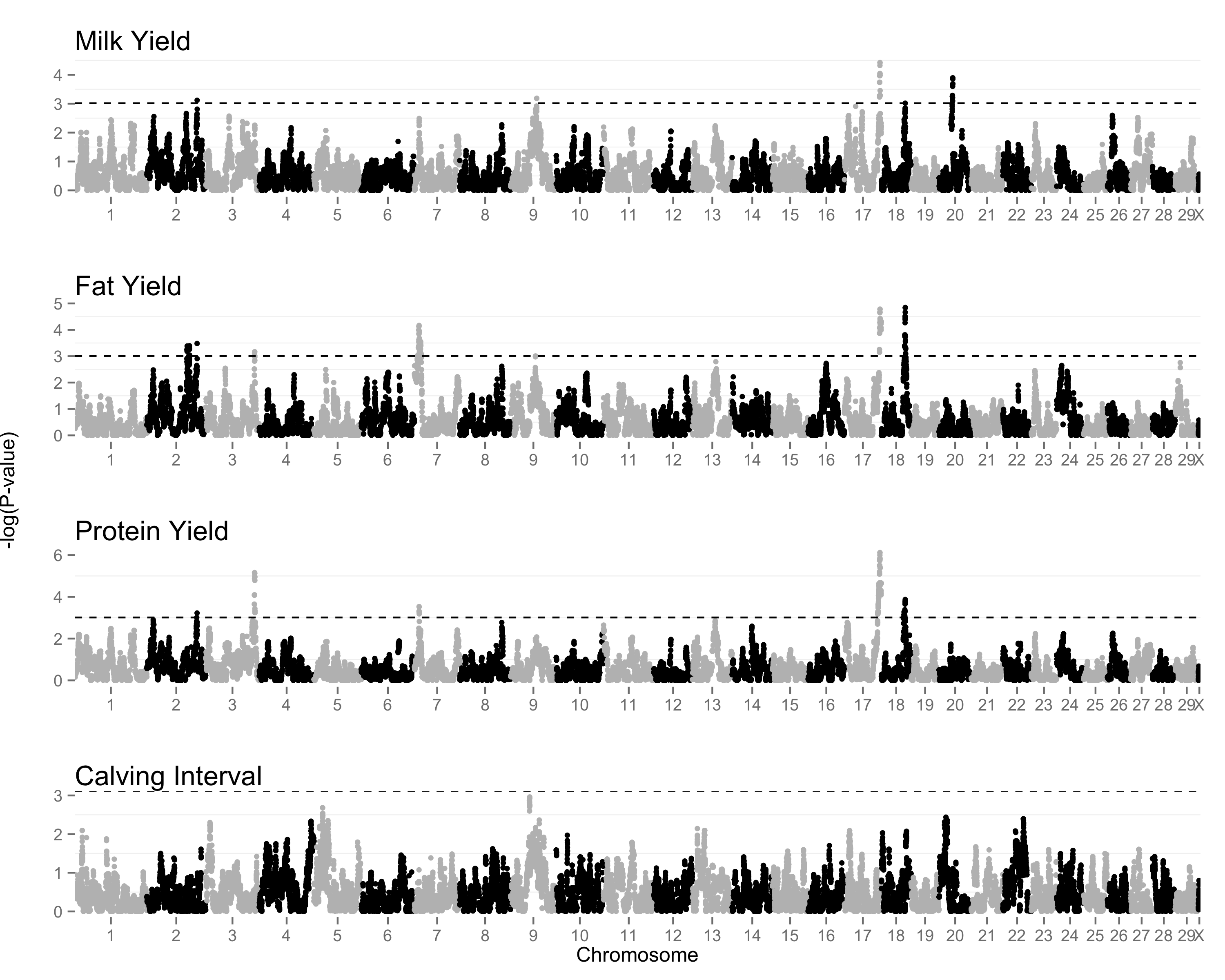


**^1^** Dotted lines represent the significance cutoff value.
