# Supplementary material for: Investigation of regions impacting inbreeding depression and their association with the additive genetic effect for United States and Australia Jersey dairy cattle
Source: BMC Genomics. 2015 Oct 19;16:813. doi: 10.1186/s12864-015-2001-7 (PMC4612420; doi:10.1186/s12864-015-2001-7)
Supplement: Additional file 3: Table S1. — Chromosomal locations and candidate genes associated with inbreeding depression for milk and fertility traits across countries. (DOC 45 kb) [file 12864_2015_2001_MOESM3_ESM.doc]

**Table S1.** Chromosomal locations and candidate genes associated with inbreeding depression for milk and fertility traits across countries.

| BTA^1^ | Location^1^ | Candidate Gene  (Location^1^) | Function | Trait^2^ |
| --- | --- | --- | --- | --- |
| 2 | 91,117,564 | ALS2  (90,664,833-90,712,586) | Motor Neuron Maintence | FY |
| 3 | 113,930,518 | UGT1A1  (113,907,720-114,031,371) | Bilirubin Metabolism | FY,PY |
| 7 | 8,860,921 | NOTCH3  (8,930,803-8,970,307) | Maintenance of Vascular  Smooth Muscle Cells | FY,PY |
| 7 | 82,173,456 | TENM2  (82,387,328-82,589,268) |  | CI |
| 7 | 96,541,131 | ANKRD32  (96,366,971-96,422,024) |  | MY,PY |
| 8 | 83,048,502 | FANCC  (83,023,629-83,270,596) | DNA Repair | FY |
| 8 | 106,817,894 | PAPPA  (107,150,317-107,379,748) |  | FY |
| 13 | 19,388,240 | PARD3  (18,982,015-19,337,608) |  | MY,PY |
| 17 | 73,118,011 | IGLL1  (72,826,680- 73,152,862) | Immune Response | MY,FY,PY |
| 18 | 52,024,379 | ETHE1  (52,048,413-52,065,7023) | Sulfide Catabolism | FY,PY |
| 19 | 14,409,010 | HNF1B  (14,287,673-14,349,579) | Insulin Regulation | FY |
| 20 | 29,322,034 | HCN1  (29,121,455-29,566,631) | Ion Channel | MY |
| 20 | 36,240,997 | LIFR  (35,917,479 – 35,966,671) | Immune Response | FY |
| 23 | 32,682,177 | ALDH5A1  (32,950,888-32,976,657) | Gamma-Amino Butyric Acid catabolism | MY,FY,PY |
| 25 | 25,450,477 | IL4R  (25,139,417-25,189,373) | Immune Response | MY |
| 25 | 29,113,430 | CALN1  (28,894,401-29,349,030) | Neuron-Specific  Calcium Binding | MY,PY |

^1^ BTA refers to chromosome and the region and location are in Mb build UMD 3.1 (http://bovinegenome.org/cgi-bin/gbrowse/bovine_UMD31/).

^2^ Traits refer to: Milk Yield (MY); Fat Yield (FY); Protein Yield (PY); Calving Interval (CI).
