# Supplementary material for: Investigation of regions impacting inbreeding depression and their association with the additive genetic effect for United States and Australia Jersey dairy cattle
Source: BMC Genomics. 2015 Oct 19;16:813. doi: 10.1186/s12864-015-2001-7 (PMC4612420; doi:10.1186/s12864-015-2001-7)

**Figure S4.** Plot of additive genomic estimated breeding (GEBV) variance, covariance between the additive genomic estimated breeding (GEBV) and ROH4Mb based genomic estimated breeding value and ROH4Mb based genomic estimated breeding value variance across the genome for fat yield on the Australian dataset. The region from 1.5 to 2.3 Mb on BTA14 were removed surrounding the DGAT mutation in order to make visualization more informative.


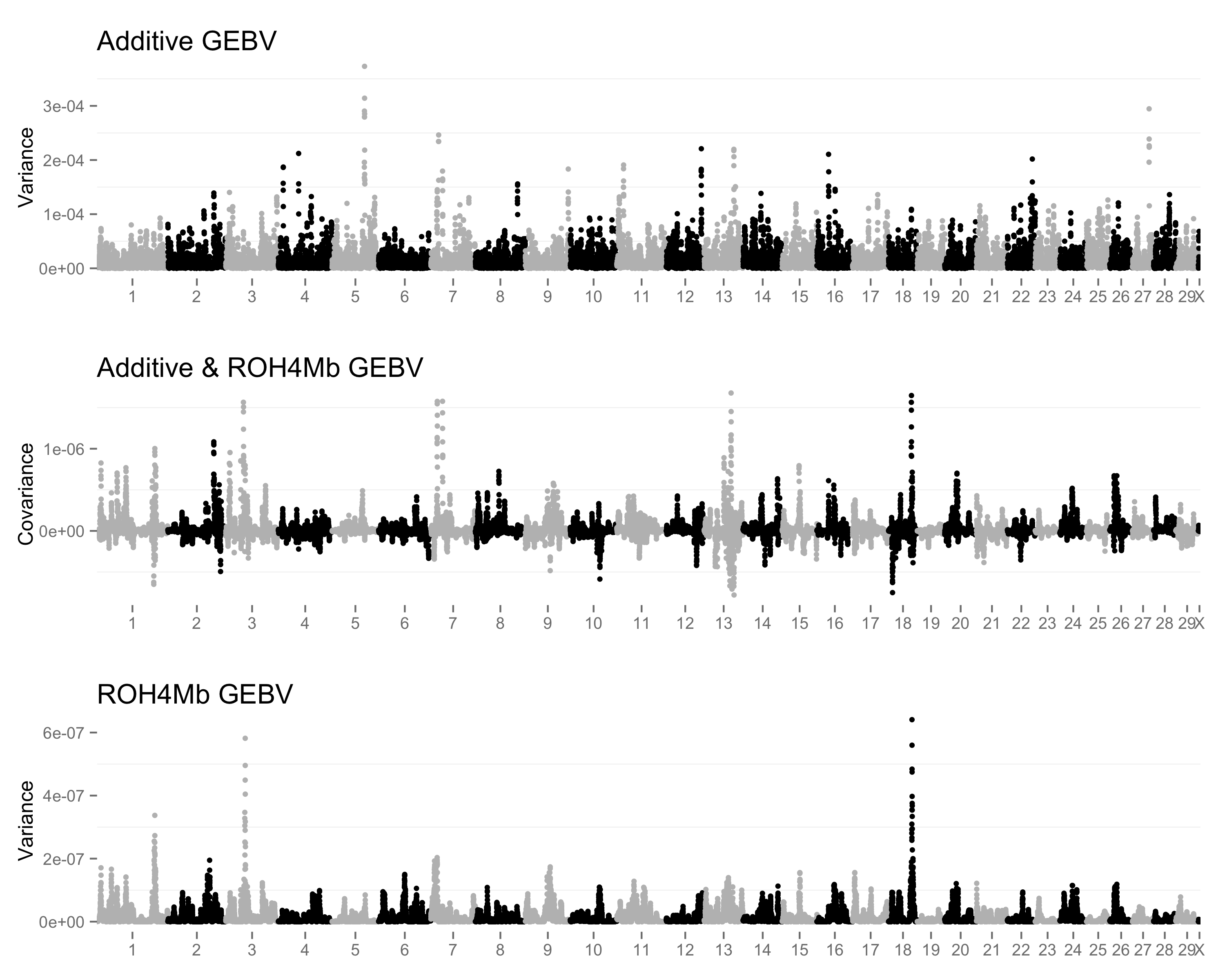

Supplement: Additional file 5: Figure S4. — Plot of additive genomic estimated breeding (GEBV) variance, covariance between the additive genomic estimated breeding (GEBV) and ROH4Mb based genomic estimated breeding value and ROH4Mb based genomic estimated breeding value variance across the genome for fat yield on the Australian dataset. The region from 1.5 to 2.3 Mb on BTA14 were removed surrounding the DGAT mutation in order to make visualization more informative. (DOC 486 kb) [file 12864_2015_2001_MOESM5_ESM.doc]
