# Supplementary material for: Investigation of regions impacting inbreeding depression and their association with the additive genetic effect for United States and Australia Jersey dairy cattle
Source: BMC Genomics. 2015 Oct 19;16:813. doi: 10.1186/s12864-015-2001-7 (PMC4612420; doi:10.1186/s12864-015-2001-7)
Supplement: Additional file 7: Figure S6. — Plot of additive genomic estimated breeding (GEBV) variance, covariance between the additive genomic estimated breeding (GEBV) and ROH4Mb based genomic estimated breeding value and ROH4Mb based genomic estimated breeding value variance across the genome for protein yield on the Australian dataset (DOC 397 kb) [file 12864_2015_2001_MOESM7_ESM.doc]

**Figure S6.** Plot of additive genomic estimated breeding (GEBV) variance, covariance between the additive genomic estimated breeding (GEBV) and ROH4Mb based genomic estimated breeding value and ROH4Mb based genomic estimated breeding value variance across the genome for protein yield on the Australian dataset

**
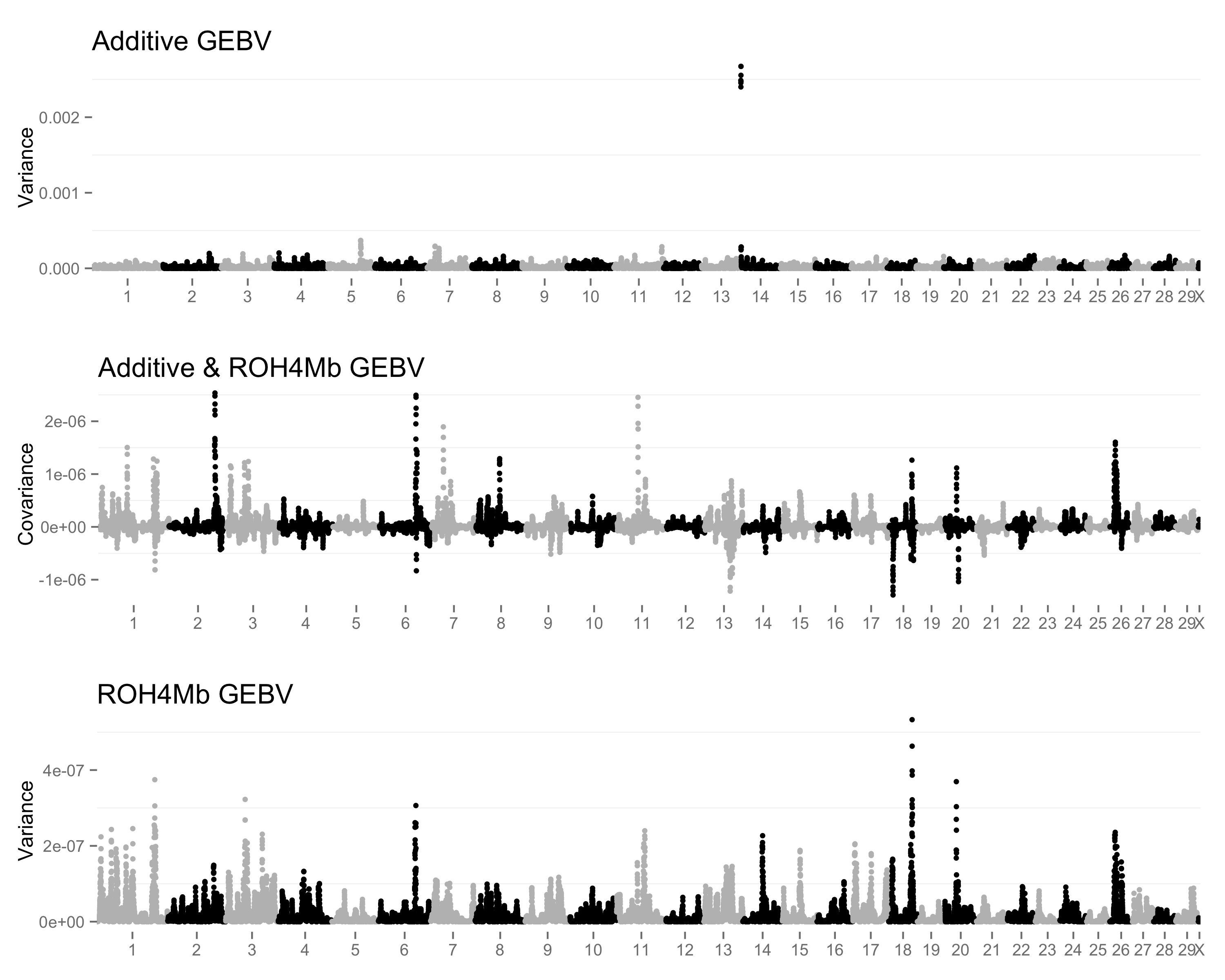
**
