# Supplementary material for: Investigation of regions impacting inbreeding depression and their association with the additive genetic effect for United States and Australia Jersey dairy cattle
Source: BMC Genomics. 2015 Oct 19;16:813. doi: 10.1186/s12864-015-2001-7 (PMC4612420; doi:10.1186/s12864-015-2001-7)
Supplement: Additional file 10: Figure S9. — Relationship between yield deviations that were corrected for the additive SNP effects estimated from the LASSO model and the residuals form the two stage analysis for protein yield and calving interval for the US population. (DOC 252 kb) [file 12864_2015_2001_MOESM10_ESM.doc]

**Figure S9.** Relationship between yield deviations that were corrected for the additive SNP effects estimated from the LASSO model and the residuals form the two stage analysis for protein yield and calving interval for the US population.

**
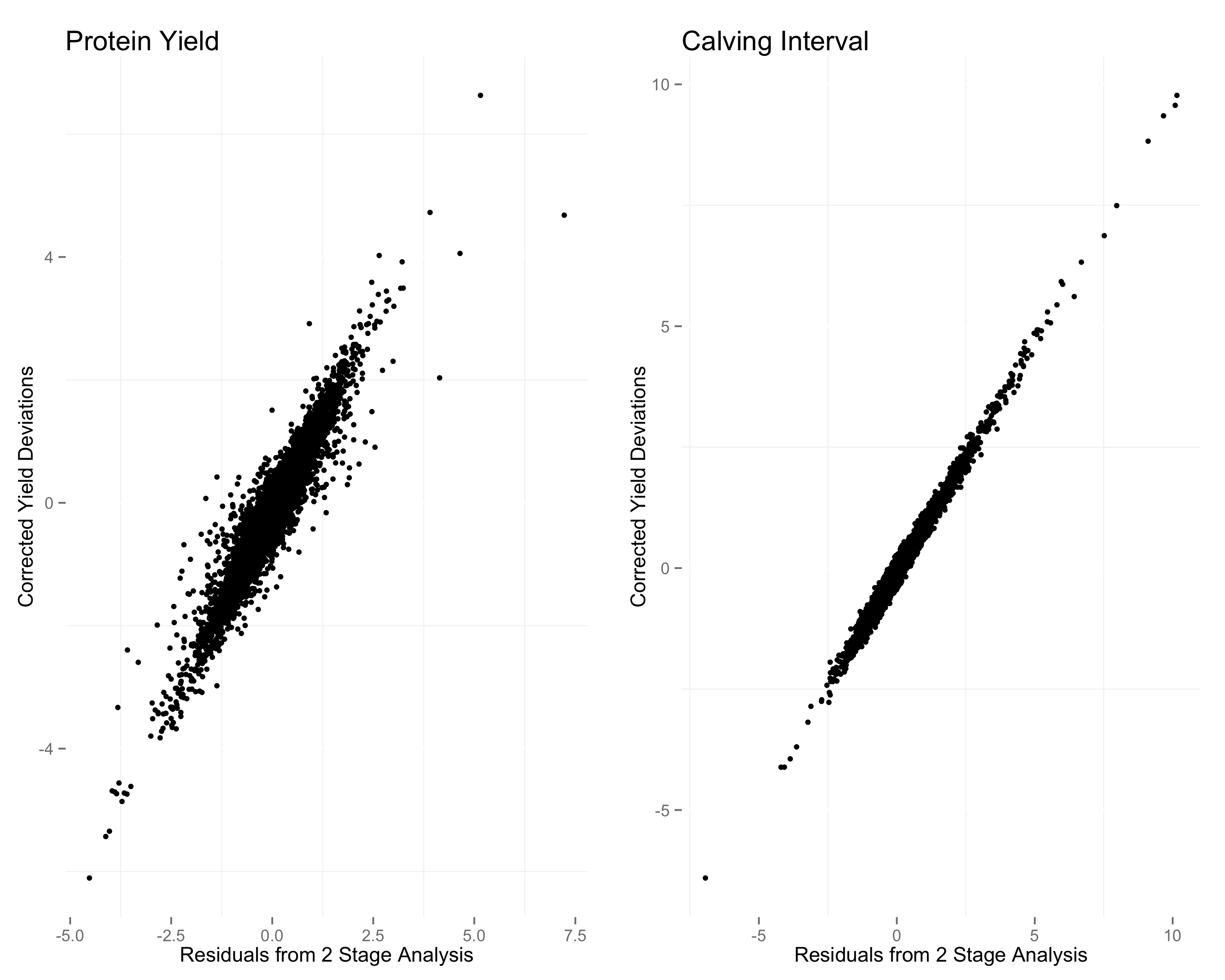
**
